# Association between triglyceride–glucose index and nonalcoholic fatty liver disease in type 2 diabetes mellitus

**DOI:** 10.1186/s12902-022-01172-7

**Published:** 2022-10-26

**Authors:** Wei Li, Yan Wang, Feng He, Zhuo Liu, Jie Dong, Yuqi Zhang, Tianfang Li, Shengyun Liu, En Chen

**Affiliations:** 1 Department of Rheumatology, The First Affiliated Hospital of Zhengzhou University, NO. 1, Jianshe East Road, 450052 Zhengzhou, Henan Province China; 2 Department of Respiratory and Critical Care Medicine, The First Affiliated Hospital of Zhengzhou University, NO. 1, Jianshe East Road, 450052 Zhengzhou, Henan Province China; 3 Department of Biochemistry and Immunology, Capital Institute of Pediatrics, NO. 2, Yabao Road, Chaoyang District, 100020 Beijing, China; 4 Pediatrics Research Institute of Hunan Province, Hunan Children’s Hospital, Changsha, China; 5 Department of Laboratory Medicine, The First Affiliated Hospital, Hengyang Medical School, University of South China, 421001 Hengyang, Hunan Province China

**Keywords:** TyG index, NAFLD, BMI, T2DM, Lipid parameters, Glycemic parameters

## Abstract

**Background:**

Lipid and glucose metabolism abnormalities are associated with nonalcoholic fatty liver disease (NAFLD). The triglyceride–glucose (TyG) index is a recently developed indicator that can identify individuals at risk for NAFLD. However, the applicability of the TyG index for identifying NAFLD in patients with type 2 diabetes mellitus (T2DM) is unclear. The aim of this study was to investigate the ability of the TyG index to identify individuals at risk for NAFLD in the T2DM population.

**Methods:**

A total of 2280 participants with T2DM were recruited in this cross-sectional study. The TyG index was calculated, and NAFLD was diagnosed by ultrasonography. Binary logistic regression models were used to evaluate the association of the TyG index, glycemic parameters and lipid parameters with NAFLD.

**Results:**

Logistic regression analysis showed that the TyG index was significantly associated with NAFLD in subjects with T2DM, the odds ratio (OR) were 3.27 (95% confidence interval [CI], 2.03–5.27; P < 0.001) for NAFLD in the highest TyG quartile after adjustment for known confounders. In stratified analysis, an elevated TyG index were more remarkably associated with NAFLD in younger patients (< 65 years; OR, 2.35; 95% CI, 1.83–3.02; P < 0.001), females (OR, 2.69; 95% CI, 1.67–4.32; P < 0.001), patients with BMI < 25 kg/m^2^ (OR, 2.80; 95% CI, 2.01–3.91; P < 0.0001), and with lower high-density lipoprotein cholesterol (< 1 mmol/L; OR, 2.76; 95% CI, 1.98–3.83; P < 0.001).

**Conclusion:**

The TyG index is significantly associated with NAFLD and shows superior ability for identify NAFLD risk compared with other lipid and glycemic parameters in T2DM.

## Background

Nonalcoholic fatty liver disease (NAFLD) has emerged as a growing global public health concern and is the most common cause of chronic liver disease worldwide[1, 2]. NAFLD is also strongly associated with overweight/obesity, insulin resistance (IR), metabolic syndrome (MS), and type 2 diabetes mellitus (T2DM)[3, 4]. NAFLD is characterized by pathological ectopic fat accumulation accompanied by low-grade chronic inflammatory state in the liver[5]. Previous studies suggested that NAFLD increases the risk of developing T2DM; worsens glycemic and lipid control; and contributes to the pathogenesis of major chronic cardiometabolic complications, such as IR, dyslipidemia, MS and cardiovascular disease (CVD), especially in people with established T2DM[6, 7].

The recently developed triglyceride–glucose (TyG) index, which is easily calculated using fasting blood glucose (FBG) and triglyceride (TG) levels, is consider an ideal substitutional marker of IR in general population[8, 9]. Additionally, the TyG index is more suitable in determining IR than other surrogate indexes, such as the Homeostatic Model Assessment for Insulin Resistance (HOMA-IR)[10, 11]. The TyG index was closely associated with BMI, total cholesterol (TC), TG, FBG, HbA1c levels, HOMA-IR, and increased incidence of MS and NAFLD[12, 13]. Furthermore, previous studies showed that glucose and lipid metabolism are closely associated with NAFLD. Lipotoxicity and glucotoxicity, which begin in hepatocyte exposure to high lipid and glucose levels, respectively, play vital roles in the development and subsequent progression of NAFLD[14]. Thus, the TyG index has been recommended as a simple and reliable indicator to identify individuals at risk for NAFLD[15, 16].

However, studies on the association between the TyG index and NAFLD and the comparison of the discriminative abilities of the TyG index, lipid parameters, and glycemic parameters for NAFLD risk, especially in patients with T2DM, are lacking[16]. Impairment of glucose and lipid metabolic pathways, which has been propelled by the prevalence of obesity and T2DM, is most likely behind the increase in NAFLD population. As the prevalence of NAFLD varies among subgroups of patients with obesity and diabetes, stratification of patients with diabetes might improve the diagnosis of NAFLD and prediction of its progression[17]. Therefore, our study aimed to characterize the relationship between the TyG index and NAFLD and compare the discriminative power of the TyG index, lipid parameters, and glycemic parameters in identifying the risk of NAFLD in patients with T2DM.

## Methods

### Study population

We included 2280 individuals with T2DM aged ≥ 18 years who had undergone liver ultrasonography. All patients included in this study were hospitalized at the First Affiliated Hospital of Zhengzhou University from January 2018 to December 2020. Subjects with previous histories of hepatic virus infections, autoimmune hepatic disease, other chronic hepatic diseases, alcohol consumption greater than 140 g/week in men and 70 g/week in women or liver cirrhosis by ultrasonography, as well as the participants without complete data on BMI, TG level, FBG level, or ultrasonic liver examination were excluded. According to the guidelines described by the Asia-Pacific Working Party, NAFLD was defined as presence of fatty liver. Fatty liver was determined by ultrasound scan, the presence of increased echogenicity of liver compared to renal cortex[18]. The study protocol was approved by the Ethical Committee of the First Affiliated Hospital of Zhengzhou University, and the requirement for informed consent was waived.

Anthropometric and biochemical measurements.

Demographics; medical history; and social habits, including smoking and drinking habits, were obtained via a self-reported questionnaire at the first visit. Height and body weight were measured, and BMI was calculated as weight divided by the square of the height (kg/m^2^). Obesity was defined according to the criteria of the Asia-Pacific region (BMI ≥ 25 kg/m^2^)[19]. Blood pressure was measured after at least 10 min of rest. Hypertension was defined as blood pressure ≥ 140/90 mmHg or the use of antihypertensive drugs. Blood samples were collected after overnight fasting and analyzed for biochemical parameters, such as aspartate aminotransferase (AST), alanine aminotransferase (ALT), gamma-glutamyl transferase (GGT), FBG, uric acid (UA), and serum lipids (including TG, TC, high-density lipoprotein cholesterol [HDL-C], and low-density lipoprotein cholesterol [LDL-C]). All measurements were determined by chemiluminescence on an auto-analyzer. Glycosylated hemoglobin (HbA1c) was measured by high-pressure liquid chromatography. The TyG index was calculated as ln [(TG (mg/dL) × FBG (mg/dL)) /2][9].

Abdominal ultrasonography.

Based on the guidelines proposed by the Asia-Pacific Working Party, NAFLD was diagnosed by the presence of fatty liver, and the presence of excessive alcohol intake (> 140 g/week for men, > 70 g/week for women) and history of hepatic viral infection and the utilization of steatogenic or hepatotoxic medicines were ruled out. Fatty liver was assessed semi-quantitatively by a professional operator using standard method as the presence or absence of hepatic steatosis by ultrasound scan and the presence of increased echo in the liver compared with the renal cortex[18].

### Statistical analyses

Normality testing was conducted, and continuous variables were presented as median and interquartile range because of their skewed distribution, whereas categorical variables were presented as percentages. Differences between the participants with NAFLD and non-NAFLD were assessed using Mann–Whitney U test for continuous variables and chi-square test for categorical variables. Binary logistic regression analysis was conducted to assess the association of the TyG index with NFALD after adjustment for confounding factors. Odds ratios (OR) with 95% confidence intervals (CI) for the risk of NAFLD were calculated. The following regression models were applied: unadjusted in Model 0; adjusted for age and sex in Model 1; further adjusted for BMI in Model 2; further adjusted for hypertension, smoking status, drinking habits, duration of diabetes mellitus, and AST and ALT levels in Model 3; and further adjusted for HbA1C, FBG, UA, TC, and HDL-C levels in Model 4. Subgroup analyses of different glycemic and lipid parameters and their interactions were performed after adjustment by Model 3. P < 0.05 was considered statistically significant. All statistical analyses were performed by SPSS version 26.0.

## Results

### Main characteristics of the study population by NAFLD.

A total of 2280 patients with T2DM were included. Among which, 67% were male, and 1543 (67.7%) had NAFLD. Compared with the non-NAFLD group, participants in the NAFLD group had a higher BMI, higher frequency of smoking or drinking, higher mean systolic and diastolic BP, shorter diabetes duration, less favorable metabolic profile (FBG, UA, AST, ALT, GGT, TC, and TG), lower HDL-C level and AST/ALT ratio, and higher TyG index (Table 1).

**Table 1:** Characteristics of the participants according to presence of NAFLD

	Non‒NAFLD N = 737 (32.3%)	NAFLD N = 1543 (67.7%)	P value
Male (%)	445 (60.4%)	1100 (71.3%)	<0.001
Age, years	53 (46‒60)	49.5 (40‒57)	<0.001
DD, years	6 (2‒12)	4 (0.5‒10)	<0.001
Hypertension (%)	316 (42.9%)	695 (45.0%)	0.33
Alcohol consumption (%)	168 (22.8%)	411 (26.6%)	0.049
Smoking (%)	127 (17.2%)	358 (23.2%)	0.001
BMI, kg/m^2^	23.7 (21.5‒25.8)	26.7 (24.5‒29.4)	<0.001
HbA1C, %	8.4 (7.1‒10.4)	8.7 (7.3‒10.4)	0.072
FBG, mmol/L	7.3 (5.8‒9.7)	7.9 (6.3‒10.5)	0.002
UA, µmol/L	268 (216‒329)	303 (254‒367)	<0.001
ALT, U/L	16 (12‒23)	21 (15‒32)	<0.001
AST, U/L	17 (14‒21)	19 (15‒24)	<0.001
GGT, U/L	17 (13‒27)	27 (18‒45)	<0.001
AST/ALT ratio	1.05 (0.82‒1.33)	0.86 (0.69‒1.08)	<0.001
SBP, mmHg	131 (121‒143)	133 (124‒144)	0.043
DBP, mmHg	81 (75‒89)	83.5 (77‒90)	<0.001
TC, mmol/L	4.15 (3.53‒4.96)	4.44 (3.76‒5.21)	<0.001
TG, mmol/L	1.21 (0.86‒1.80)	1.9 (1.3‒3.03)	<0.001
HDL-C, mmol/L	1.14 (0.93‒1.39)	1.0 (0.82‒1.19)	<0.001
LDL-C, mmol/L	2.5 (1.96‒3.20)	2.6 (1.96‒3.27)	0.117
TyG index	8.89 (8.44‒9.44)	9.42 (8.93‒9.98)	<0.001

Data are presented as median (interquartile range) or percentage

ALT: alanine transferase; AST: aspartate transferase; BMI: body mass index; SBP: systolic blood pressure; DBP: diastolic blood pressure; DD: Diabetes Duration; FBG: fasting blood glucose; GGT: gamma-glutamyl transferase; HbA1c: glycosylated hemoglobin; HDL-C: high-density lipoprotein cholesterol; LDL-C: low-density lipoprotein cholesterol; NAFLD: nonalcoholic fatty liver disease; TC: high cholesterol; TG: triglyceride; TyG: triglycerides and glucose index; UA: uric acid

Associations between the TyG index and NAFLD risk.

**Fig. 1:**
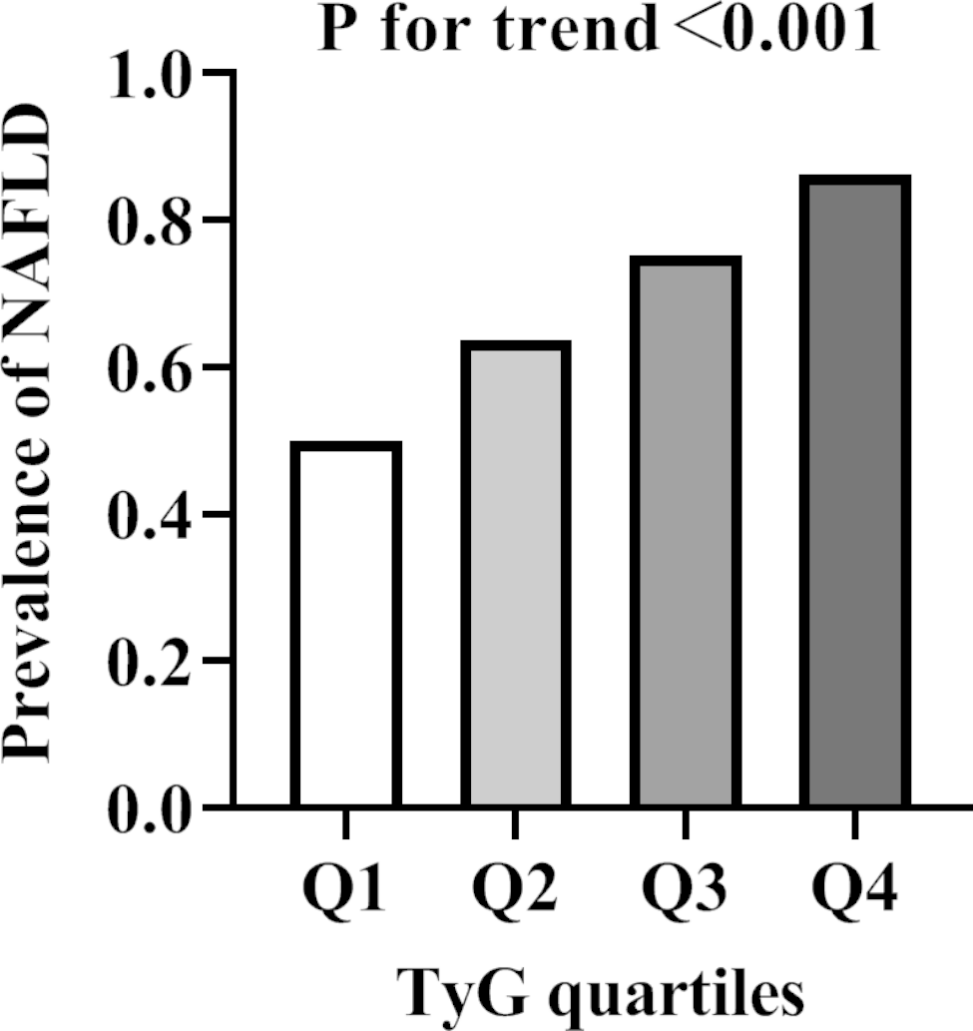
Prevalence of NAFLD in the quartiles of TyG. Prevalence of NAFLD according to the quartiles of TyG: Q1 (50%), Q2 (64%); Q3 (75%), Q4 (86%)

The prevalence of NAFLD remarkably increased with the increase in TyG level. Binary logistic regression was performed to examine whether TyG index is independently associated with NAFLD in patients with T2DM within four different models. The NAFLD risk significantly increased with the increasing TyG level, the OR were 5.0 in the highest TyG quartile (95% CI, 3.79–6.60; P < 0.001; Model 0). As presented in Table 2, the OR for NAFLD were higher with increasing TyG quartiles after adjusting for age, gender (4.24, 95% CI 3.20–5.63, P < 0.001 for trend; Model 1). In the highest TyG quartile, the OR were 3.02 (95% CI, 2.22–4.11; P < 0.001 for trend) for NAFLD after further adjustment for BMI (Model 2), and the OR were 2.90 (95% CI, 2.12–3.98; P < 0.001) for NAFLD after adjustment for Model 3. The associations persisted after additional adjustment in Model 4 (OR, 3.27; 95% CI, 2.03–5.27; P < 0.001). In addition, the prevalence of NAFLD in individuals with the highest TyG quartile was 86.3%, which showed a 1.73-fold increase compared with the prevalence in the lowest quartile (50%, Fig. 1).

**Table 2:** Odds ratios for NAFLD in different quartiles of TyG index

	Q1 (Reference)	Q2 OR (95% CI)	Q3 OR (95% CI)	Q4 OR (95% CI)	P value
TyG index	<8.76	8.76‒9.28	9.28‒9.85	≥ 9.85	‒
Unadjusted	1	1.76 (1.38‒2.23)	2.99 (2.32‒3.85)	5.00 (3.79‒6.60)	<0.001
Model 1	1	1.74 (1.36‒2.21)	2.93 (2.27‒3.79)	4.24 (3.20‒5.63)	<0.001
Model 2	1	1.42 (1.09‒1.85)	2.45 (1.85‒3.24)	3.02 (2.22‒4.11)	<0.001
Model 3	1	1.37 (1.05‒1.79)	2.54 (1.91‒3.39)	2.90 (2.12‒3.98)	<0.001
Model 4	1	1.46 (1.08‒1.97)	2.76 (1.93‒3.96)	3.27 (2.03‒5.27)	<0.001

Model 0 was unadjusted

Model 1 was adjusted for age, sex

Model 2 was adjusted for age, sex and BMI

Model 3 was adjusted for all variables in model 2 plus hypertension, smoking status, drinking habits, duration of diabetes mellitus, and AST and ALT levels

Model 4 was adjusted for all variables in model 3 plus HbA1C, FBG, UA, TC, and HDL-C levels

Association of the TyG index, glycemic parameters, and lipid parameters with NAFLD.

Binary logistic regression models that separately consider each glycemic and lipid parameters as predictors of NAFLD were performed. Table 3 shows the OR and 95% CI of NAFLD with FBG, HbA1c, BMI, TC, TG, HDL-C, LDL-C, TyG index, TG/HDL-C ratio, and AST/ALT ratio in the total population within Model 3. As shown in Table 3, BMI (OR, 1.31; 95% CI, 1.26–1.35; P < 0.001), TC (OR, 1.13; 95% CI, 1.03–1.23; P = 0.009), TG (OR, 1.25; 95% CI, 1.15–1.34; P < 0.001), HDL-C (OR, 0.41; 95% CI, 0.29–0.57; P < 0.001), TyG index (OR, 1.82; 95% CI, 1.58–2.10; P < 0.001), TG/HDL-C (OR, 1.14; 95% CI, 1.08–1.20; P < 0.001), and AST/ALT ratio (OR, 0.68; 95% CI, 0.55–0.85; P < 0.001) were associated with NAFLD. However, FBG, HbA1C, and LDL-C levels had no association with NAFLD risk as observed in Model 3. Although the OR of HDL-C for NAFLD risk is higher in Model 3, the OR dramatically decreased after further adjustment in Model 4 (OR, 0.52; 95% CI, 0.32–0.84; P = 0.008). The OR of TyG stood out the most in comparison with the ORs of lipid and glycemic parameters, which indicates that TyG index can be a better discriminator of NAFLD.

**Table 3:** Association of the TyG index, glycemic, lipid parameters with NAFLD in total subjects

	OR (95% CI)	P value
TyG index	1.82 (1.58‒2.10)	<0.001
BMI, kg/m^2^	1.31 (1.26‒1.35)	<0.001
FBG, mmol/L	1.03 (0.99‒1.06)	0.1
HbA1c, %	1.05 (0.99‒1.10)	0.06
ALT, U/L	1.02 (1.01‒1.04)	<0.001
AST/ALT ratio	0.68 (0.55‒0.85)	<0.001
TC, mmol/L	1.13 (1.03‒1.23)	0.009
TG, mmol/L	1.25 (1.15‒1.34)	<0.001
HDL-C, mmol/L	0.41 (0.29‒0.57)	<0.001
LDL-C, mmol/L	1.04 (0.93‒1.15)	0.519
TG/HDL-C ratio	1.14 (1.08‒1.20)	<0.001

ALT: alanine transferase; AST: aspartate transferase; BMI: body mass index; FBG: fasting blood glucose; HbA1c: glycosylated hemoglobin; HDL-C: high-density lipoprotein cholesterol; LDL-C: low-density lipoprotein cholesterol; TG: triglyceride; TC: high cholesterol; TyG: triglycerides and glucose index

Adjusted for age, sex, BMI, hypertension, smoking status, drinking habits, duration of diabetes mellitus, AST, and ALT levels

Associations between the TyG index and NAFLD in subgroups of age, sex, BMI, HDL-C, and AST/ALT ratio.

Stratified analyses were conducted in different subgroups (age, sex, BMI, HDL-C, and AST/ALT ratio) to further validate the above results after adjustment for Model 4 as shown in Table 4. The results suggested that compared with participants with lower TyG, higher TyG levels were more remarkably associated with the risk of NAFLD in younger age (< 65 years; OR, 2.35; 95% CI, 1.83–3.02; P < 0.001), female (OR, 2.69; 95% CI, 1.67–4.32; P < 0.001), lower BMI (< 25 kg/m^2^; OR, 2.80; 95% CI, 2.01–3.91; P < 0.001), lower HDL-C (< 1 mmol/L; OR, 2.76; 95% CI, 1.98–3.83; P < 0.001), and higher AST/ALT ratio (≥ 0.9; OR, 2.63; 95% CI, 1.91–3.62; P < 0.001). However, no association between TyG index and NAFLD was observed in the older age subgroups (≥ 65 years; OR, 1.49; 95% CI, 0.65–3.39; P = 0.343). Additionally, in the subgroup analysis, the TyG index had interaction effects with age (P value for interaction = 0.043), BMI (P value for interaction < 0.001), HDL-C (P value for interaction < 0.001), and AST/ALT (P for interaction = 0.011) on NAFLD risk after adjusting for potential confounders (Model 4). The result indicates an excess risk due to the additive interaction.

**Table 4:** Subgroup analysis of the association between TyG index and NAFLD

	Stratified Group	OR (95% CI)	P value	P for interaction
Age, years	<65	2.35 (1.83‒3.02)	<0.001	0.043
	≥ 65	1.49 (0.65‒3.39)	0.343	
Sex	Female	2.69 (1.67‒4.32)	<0.001	0.486
	Male	2.09 (1.59‒2.76)	<0.001	
BMI, kg/m^2^	<25	2.80 (2.01‒3.91)	<0.001	<0.001
	≥ 25	1.86 (1.33‒2.60)	<0.001	
HDL-C, mmol/L	<1	2.76 (1.98‒3.83)	<0.001	<0.001
	≥ 1	2.26 (1.65‒3.11)	<0.001	
AST/ALT ratio	<0.9	1.63 (1.13‒2.34)	0.009	0.011
	≥ 0.9	2.63 (1.91‒3.62)	<0.001	

BMI: body mass index; ALT: alanine transferase; AST: aspartate transferase; HDL-C: high-density lipoprotein cholesterol; TyG: triglycerides and glucose index

Adjusted for age, sex, BMI, hypertension, smoking status, drinking habits, duration of diabetes mellitus, AST and ALT levels, HbA1C, FBG, UA, TC, and HDL-C levels

## Discussion

Although most studies explored the relationship between the TyG index and NAFLD risk, these studies focused on the general population[15, 20, 21]. Our present study focused on the associations of the TyG index, glycemic parameters, and lipid parameters with NAFLD in a T2DM population. This cross-sectional study observed a strong and positive association between the TyG index and NAFLD risk after adjustment for potential confounders. HDL-C, TG, BMI, ALT, and AST/ALT ratio were also associated with NAFLD risk but inferior to the TyG index. Further stratification analysis showed that females, patients with lesser BMI (< 25 kg/m^2^), younger age (< 65 years), and lower HDL-C level (< 1 mmol/L) have higher NAFLD risks when the TyG index is high. Therefore, based on the above observations, the TyG index can be used as an effective predictor of NAFLD risk in T2DM population.

NAFLD is closely associated with obesity and MS and characterized by excessive lipid accumulation in the liver tissue, which leads to hepatic IR[5, 22]. Furthermore, the hepatic IR subsequently leads to the overproduction of FBG and low-density lipoprotein. Lipotoxicity and glucotoxicity play central roles in the development and progression of NAFLD[14]. Our results showed that individuals with NAFLD had considerably higher BMI, FBG, TC, and TG levels than those without NAFLD. IR is a crucial pathophysiological mechanism of MS, which is a metabolic risk factor associated with an increased risk for T2DM and NAFLD[6, 23]. IR identification is deemed helpful to stratify and support the personalized treatment of patients with NAFLD. The TyG index combined with TG and FBG levels has been proposed as an effective substitute for IR[9]. A recent cross-sectional survey showed that the TyG index is significantly associated with hypertension, and shows the superior discriminative ability for hypertension compared with lipid parameters and glycemic parameters[24]. The TyG index is applied in assessing the value of TG and FBG because of the two parameters are intensively related. Hypertriglyceridemia is the most prevalent abnormalities in patients with T2DM, and its association with the risk of CVDs (including hypertension and NAFLD) has been clearly demonstrated. In line with previous study, the present study demonstrated the associations of BMI, TC, TG, HDL-C, and TyG with NAFLD risk, however, the association of the TyG index with NAFLD was stronger than those of lipid or glycemic parameters. Thus, the TyG index is a better indicator for identifying NAFLD risk compared with other lipid and glycemic parameters in patients with T2DM.

Studies on the interaction between BMI and HDL-C, sex, or age in NAFLD are limited. The interaction between HDL-C, age and TyG index might be due to the prevalence of low HDL-C in younger groups[25]. Thus, our study demonstrated that female, younger age (< 65 years), and low HDL-C level (< 1 mmol/L) are associated with higher NAFLD risks when the TyG index is higher. The effect of hormone levels on glucose, lipid metabolism and IR, might account for this discrepancies in different sexes and thus requires further explore[26, 27]. Although the LDL-C level was considered to be the most crucial lipid risk factor and therapeutic goal for CVDs[28], however, there was no significant association of LDL-C with the risk of NAFLD in the present study. Furthermore, when HDL-C and TC were adjusted in Model 4, the TyG index was still remarkably associated with NAFLD risk in our study. The precise mechanism of the association between TyG index and NAFLD has not been fully elucidated, but it is thought to involved IR, endothelial dysfunction, systemic inflammation, glucose and lipid metabolism disorders[29].

In our present study, we examined the association of the TyG index, glycemic parameters, and lipid parameters with NAFLD. Obviously, in all participants, the OR of TyG stood out the most in comparison with the ORs of other lipid and glycemic parameters, which indicates that the TyG index might be superior to other glycemic or lipid parameters in associating NAFLD risk. The TyG index is applied in evaluating the levels of FBG and TG in T2DM population due to the two parameters are closely interrelated. High TG levels remains one of the most prevalent abnormalities in T2DM patients, and its association with increased risk of NAFLD has been fully demonstrated[30]. This study revealed that the TyG index helps to identify potential risks of NAFLD in individuals who would otherwise be neglected. Thus, clinicians should be put their attention to individuals with high FBG and TG levels.

Serum ALT level is commonly used as a surrogate indicator for evaluating liver function in various liver diseases, and ALT seems to be closely associated with steatohepatitis[31, 32]. Furthermore, ALT has high specificity for liver injury and is considered as a cardiometabolic risk factor associated with IR, MS, and CVD[33, 34]. In accordance with prior studies, our findings showed that only 18.8% of individuals with NAFLD had increased ALT levels (≥ 40 U/L)[35, 36]. This result implicated that elevated ALT is probably inadequate to evaluate the NAFLD risk of individuals with T2DM. Thus, more sensitive biomarkers for predict the risk of NAFLD are needed. Furthermore, our data demonstrated that the diagnostic accuracy of the TyG index was superior to those of ALT and AST/ALT ratio in identifying NAFLD risk in patients with T2DM.

Obesity leads to the development of MS and comorbidities, including hypertension, hyperlipidemia, IR, NAFLD, T2DM, and CVD[37, 38]. Obesity seems to play a crucial role in the initial development and progression of NAFLD[3, 39]. Previous studies found that NAFLD prevalence increases almost linearly with BMI, whereas obesity is independently related to NAFLD irrespective of other metabolic factors[40]. Another study showed that the association of TyG and NAFLD risk was significantly stronger in non-obese subjects than that in obese ones[20]. Thus, the predictive power of TyG index for NAFLD risk was partially affected by BMI of the individuals. Interestingly, our present study also demonstrated that the OR of NAFLD dramatically decreased after adjustment for BMI in Model 2 (OR, 4.24 vs. 3.02). Furthermore, subgroup analysis indicated that the relationship between the TyG index and NAFLD risk was significantly stronger in non-obese subjects than in obese ones (OR, 2.80 vs. 1.86). Previous study demonstrated that subjects who are of normal weight but metabolically unhealthy have a higher risk of fatty liver and CVD, compared to normal weight and metabolically healthy population. The existence of a distinct fat distribution and lipodystrophy in these population may be account for the potential mechanism[41, 42]. Therefore, these results suggested that BMI is an important factor that affects the efficacy of the TyG index in identifying individuals with NAFLD risk[43].

Nevertheless, several limitations should be noted in the present study. First, the NAFLD diagnosis was only based on ultrasonography rather than liver biopsy. The accuracy and sensitivity of NAFLD diagnosis may not be absolutely reliable. However, ultrasound examination for the diagnosis of NAFLD is a preferable and accessible imaging method in clinical practice[44]. Furthermore, our study population included only T2DM patients. The conclusion may not be applicable to the general population. Therefore, identifying the causal relationship between the TyG index and NAFLD in larger and more various population is necessary.

## Conclusion

The results of present study revealed a remarkable association between the TyG index and NAFLD in patients with T2DM, and the TyG index is superior to HbA1c, FBG, AST/ALT ratio, and other lipid parameters in determining NAFLD risk. Therefore, we suggest that the TyG index could be a more efficient, useful, and simple indicator for the screening and management of NAFLD in T2DM population.

## Data Availability

The datasets in the current study are available from the corresponding author on reasonable request.

## List of abbreviations

ALT: Alanine transferase
AST: Aspartate transferase
BMI: Body mass index
CVD: Cardiovascular disease
CI: Confidence interval
DBP: Diastolic blood pressure
eGFR: Estimated glomerular filtration rate
FBG: Fasting plasma glucose
GGT: Gamma-glutamyl transferase
HbA1c: Glycosylated hemoglobin
HDL-C: High-density lipoprotein cholesterol
IR: Insulin resistance
LDL-C: Low-density lipoprotein cholesterol
MS: Metabolic syndrome
NAFLD: Non-alcoholic fatty liver disease
OR: Odds ratio
SBP: Systolic blood pressure
TC: Total cholesterol
TG: Triglyceride
TyG: Triglyceride and glucose index
T2DM: Type 2 diabetes mellitus
UA: Uric acid
